# Association of atherogenic index of plasma and its modified indices with gestational diabetes mellitus: a prospective cohort study based on the Korean population

**DOI:** 10.3389/fendo.2026.1923064

**Published:** 2026-08-27

**Authors:** Feng Lu, Yingjie Chen

**Affiliations:** 1 Department of Anesthesiology, The Second Affiliated Hospital of Nanchang University, Nanchang, Jiangxi, China; 2 Department of Emergency, Shishi Municipal Hospital, Quanzhou, Fujian, China

**Keywords:** AIP-BMI, AIP-WC, atherogenic index of plasma (AIP), gestational diabetes mellitus (GDM), risk prediction

## Abstract

**Background:**

Gestational diabetes mellitus (GDM) is a common pregnancy complication, yet current diagnosis at 24–28 weeks may occur after metabolic changes have already begun. The atherogenic index of plasma (AIP, log₁₀[TG/HDL-C]) has shown promise for early GDM risk stratification, but it does not incorporate obesity-related parameters. We evaluated whether first-trimester AIP and its modified indices, AIP-BMI and AIP-WC, are associated with subsequent GDM and compared their predictive performance.

**Methods:**

This secondary analysis of a prospective Korean cohort included 581 women with singleton pregnancies. AIP, AIP-BMI (AIP × BMI), and AIP-WC (AIP × waist circumference) were calculated using fasting measurements obtained at 10–14 weeks of gestation; BMI and waist circumference were algebraically reconstructed from the full-precision components of the hepatic steatosis index and fatty liver index because contemporaneous direct measurements were unavailable in the public dataset. GDM was diagnosed at 24–28 weeks using the two-step ACOG approach. Multivariable logistic regression, restricted cubic spline, sensitivity, subgroup, and receiver operating characteristic analyses were performed.

**Results:**

GDM occurred in 36 women (6.2%). In the fully adjusted model, AIP (per 0.1-unit increase: OR 1.66, 95% CI 1.36–2.03; Q3 vs Q1: OR 7.86, 95% CI 1.93–32.08), AIP-BMI (OR 1.20, 95% CI 1.11–1.31), and AIP-WC (OR 1.05, 95% CI 1.03–1.08) were associated with GDM (all P < 0.001). Overall associations were significant in restricted cubic spline analyses, without evidence of nonlinearity. Among individual indices, AIP-BMI had the largest apparent AUC (83.05%, sensitivity 81%, specificity 80%), followed by AIP-WC (81.32%) and AIP (78.66%). The AIP + AIP-BMI model had the largest apparent AUC among evaluated models (84.19%, sensitivity 81%, specificity 81%). Results remained broadly consistent in sensitivity analyses excluding participants with NAFLD, whereas subgroup analyses were underpowered to detect interactions.

**Conclusion:**

First-trimester AIP, AIP-BMI, and AIP-WC were associated with subsequent GDM, and AIP-BMI showed higher apparent discrimination than AIP alone in this sample. These findings require confirmation in larger cohorts with directly measured anthropometry and appropriate internal and external validation.

## 1 Introduction

Gestational diabetes mellitus (GDM) is a pregnancy complication characterized by glucose intolerance that is first detected during gestation, representing one of the most frequently encountered medical disorders in obstetric practice (1, 2). Driven by rising obesity rates among women of childbearing age, later childbearing, and shifts in lifestyle patterns, the worldwide prevalence of GDM has shown a consistent upward trajectory (3, 4). Beyond its well-documented association with immediate adverse perinatal outcomes—including fetal macrosomia, elevated rates of cesarean delivery, and neonatal hypoglycemia—GDM also confers lasting metabolic risks, predisposing both mothers and their offspring to future type 2 diabetes, obesity, and cardiovascular disease (5, 6).

Current clinical guidelines recommend routine GDM screening between 24 and 28 weeks of gestation using the oral glucose tolerance test (OGTT) (7). However, a growing body of evidence suggests that by this gestational window, potentially irreversible changes—including fetal metabolic programming alterations and maternal pancreatic β-cell dysfunction—may have already been set in motion (8, 9). This temporal gap between the onset of pathophysiological changes and clinical diagnosis highlights the critical need for risk identification earlier in pregnancy. Therefore, identifying simple, cost-effective biomarkers applicable in the first trimester has become an important research priority for early GDM prediction.

The atherogenic index of plasma (AIP), calculated as log_10_(TG/HDL-C), has emerged as a lipid-based biomarker indicative of lipoprotein particle size distribution and systemic atherosclerotic risk (10). Prior work has consistently demonstrated significant associations between AIP and type 2 diabetes (T2DM), prediabetes, and cardiometabolic conditions (11–13). In a prospective Korean cohort, Zhang et al. (14) reported that for each 0.1-unit increment in AIP measured at 10–14 gestational weeks, GDM risk rose by 58%, with those in the highest AIP tertile exhibiting an adjusted OR of 5.76 (95% CI: 2.18–15.23) relative to the lowest tertile.

Despite its utility, AIP is derived exclusively from lipid parameters (TG and HDL-C) and does not incorporate obesity-related anthropometric measures—a notable limitation given that obesity, particularly central obesity, is a well-recognized GDM risk factor that operates synergistically with dyslipidemia in the pathogenesis of insulin resistance (15, 16). Recently, Wang et al. developed modified AIP indices by integrating AIP with waist circumference (WC), waist-to-height ratio (WHtR), and body mass index (BMI) among individuals with cardiovascular-kidney-metabolic syndrome, demonstrating that AIP-WC and AIP-WHtR outperformed AIP alone in predicting stroke (17). To date, however, the predictive value of these modified AIP indices for GDM has not been evaluated.

Against this background, the present study sought to: (1) examine the independent associations of first-trimester AIP and its modified indices (AIP-BMI and AIP-WC) with subsequent GDM risk; and (2)assess and compare the predictive performance of these indices, both individually and in combination, using prospectively collected data from a Korean pregnancy cohort, with the overarching goal of providing a practical, low-cost screening tool for early GDM identification.

## 2 Methods

### 2.1 Study design and data source

This investigation represents a secondary analysis of data from a prospective cohort study conducted at two tertiary referral centers in South Korea—Incheon Seoul Women’s Hospital and Seoul National University Boramae Medical Center—between November 2014 and July 2016 (ClinicalTrials.gov identifier: NCT02276144) (18). The dataset is publicly accessible under a Creative Commons Attribution License. Ethical approval was secured by the original study team (19), and each participant provided written informed consent prior to enrollment.

### 2.2 Study population

The parent study enrolled 663 women with singleton gestations, excluding those with chronic liver disease, heavy alcohol intake, pre-existing diabetes, or initial presentation after 14 weeks of gestation. After 40 women were excluded due to loss to follow-up or delivery before 34 weeks, 623 participants remained.

**Figure 1:**
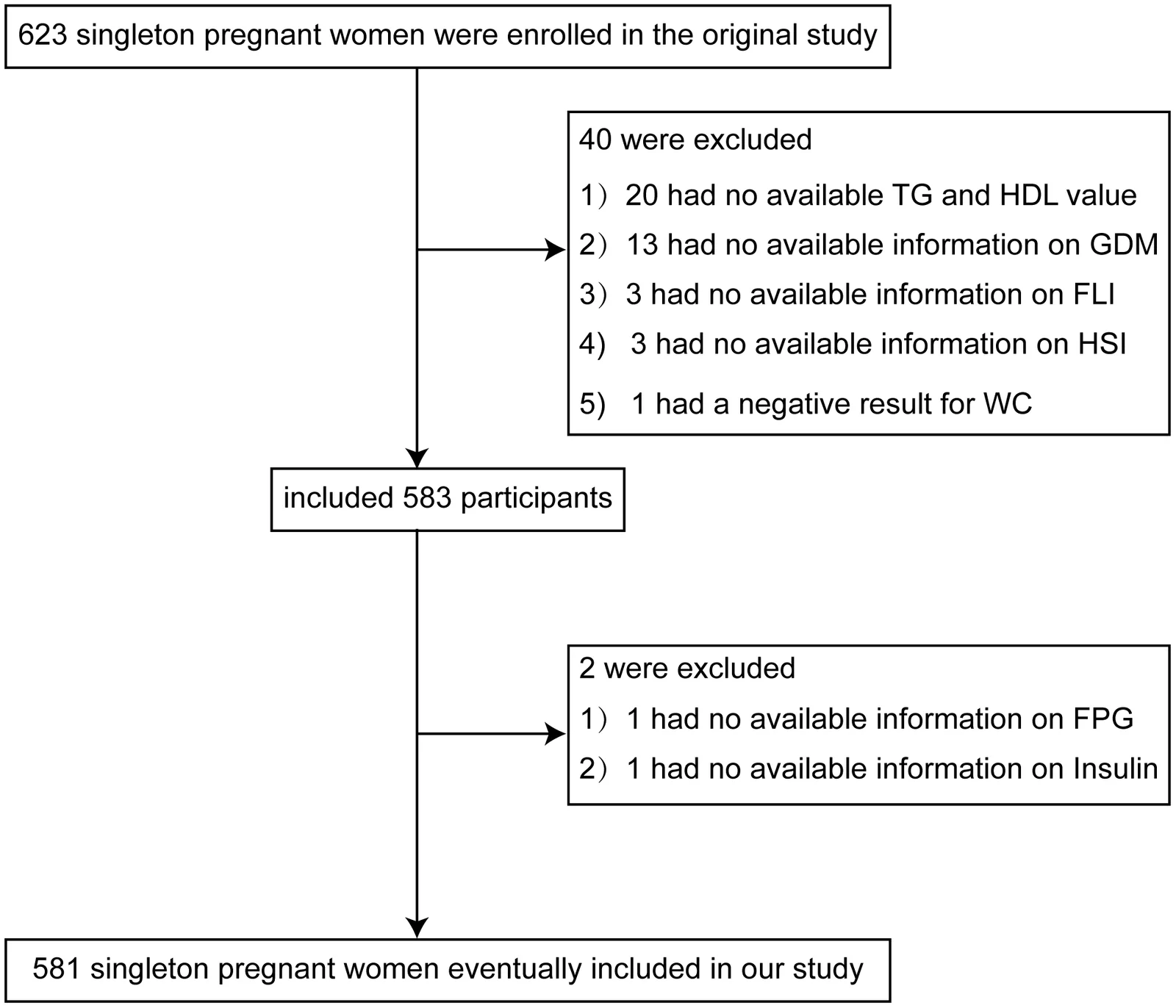
Flowchart of participant selection. The non-positive formula-derived WC was 0 cm (participant No. 602).

For the current analysis, we excluded 20 participants without TG or HDL-C data, 13 without an available GDM classification, 6 without the index components required to reconstruct BMI or WC (3 with missing HSI and 3 additional participants with missing FLI), 1 participant with a non-positive formula-derived WC (0 cm; participant No. 602), and 2 participants without fasting glucose or insulin data. The final analytic sample comprised 581 women, of whom 36 (6.2%) developed GDM (**Figure 1**). Baseline characteristics did not differ significantly between included and excluded participants (**Supplementary Table 1**).

### 2.3 Data collection

Trained study personnel obtained and compiled all data. General demographic and clinical characteristics—including maternal age, nulliparity, pre-pregnancy body mass index (preBMI), alcohol consumption, and diabetes history—were gathered via standardized questionnaires. Fasting venous blood specimens (≥8-hour fast) were collected at 10–14 gestational weeks to quantify the following biochemical parameters: aspartate aminotransferase (AST), alanine aminotransferase (ALT), gamma-glutamyl transferase (GGT), total cholesterol (TC), triglycerides (TG), high-density lipoprotein cholesterol (HDL-C), low-density lipoprotein cholesterol (LDL-C), fasting plasma glucose (FPG), and insulin. The homeostasis model assessment of insulin resistance (HOMA-IR) was derived as [FPG (mmol/L) × insulin (μIU/mL)]/22.5. NAFLD was identified through hepatic ultrasound examination. Further details regarding data collection and variable definitions are available in the original study (19).

### 2.4 Exposure variables

The primary exposures were AIP and its modified indices, calculated from fasting measurements obtained at 10–14 weeks of gestation. Consistent with the source AIP analysis and the modified-index definition, AIP was operationalized as log_10_[TG (mg/dL)/HDL-C (mg/dL)]. The modified indices were AIP-BMI = AIP × BMI (kg/m²) and AIP-WC = AIP × WC (cm) (17). AIP tertiles were Q1 (<0.16), Q2 (0.16–0.32), and Q3 (≥0.32).

Directly measured BMI and WC at 10–14 weeks were not available in the public secondary dataset. We therefore solved for these quantities from the recorded full-precision components of the HSI and FLI equations (**Supplementary Table 2**), rather than from rounded published summary values. Because all participants were women and pre-existing diabetes was an exclusion criterion, BMI was calculated as BMI = HSI − 8 × (ALT/AST) − 2. WC was calculated as WC = {ln[FLI/(100 − FLI)] − 0.953 × ln(TG) − 0.139 × BMI − 0.718 × ln(GGT) + 15.745}/0.053. No intermediate value was rounded. Participant No. 602 yielded a formula-derived WC of exactly 0 cm and was treated as invalid and excluded before analysis.

We assessed numerical stability using first-order error propagation. The relevant derivatives were ∂WC/∂TG = −0.953/(0.053 × TG), ∂WC/∂GGT = −0.718/(0.053 × GGT), ∂WC/∂BMI = −0.139/0.053, and ∂WC/∂FLI = 100/[0.053 × FLI × (100 − FLI)], with FLI expressed in percentage points. At the analytic-sample medians (TG 110 mg/dL, GGT 12 IU/L, and FLI 11.52%), these were −0.163 cm per mg/dL, −1.129 cm per IU/L, −2.623 cm per BMI unit, and 1.852 cm per FLI percentage point, respectively. A simultaneous ±5% perturbation in TG and GGT produced a root-sum-square WC shift of approximately 1.13 cm. Because FLI and HSI were stored at full precision and unrounded BMI was used, arithmetic rounding of these intermediate quantities was negligible.

### 2.5 Outcome variable

The diagnosis of GDM was established between 24 and 28 gestational weeks using the two-step screening algorithm recommended by the American College of Obstetricians and Gynecologists (ACOG). In the first step, all participants underwent a 50-g glucose challenge test (GCT). Those with a 1-h plasma glucose level ≥ 7.8 mmol/L proceeded to the second step, a diagnostic 100-g oral glucose tolerance test (OGTT). GDM was diagnosed if at least two of the four Carpenter–Coustan plasma glucose thresholds were met or exceeded: fasting ≥ 5.3 mmol/L (95 mg/dL), 1-h ≥ 10.0 mmol/L (180 mg/dL), 2-h ≥ 8.6 mmol/L (155 mg/dL), and 3-h ≥ 7.8 mmol/L (140 mg/dL). Women with a GCT value < 7.2 mmol/L were classified as non-GDM.

### 2.6 Covariates

Covariate selection was informed by clinical reasoning and established GDM risk factors (18) and was refined using least absolute shrinkage and selection operator (LASSO) regression to limit model complexity given the 36 GDM events. The selected covariates were age, preBMI, nulliparity, ALT, GGT, HOMA-IR, TC, LDL-C, and NAFLD. Variance inflation factors were <5 (**Supplementary Table 3**). Although LASSO reduced the candidate set, it did not eliminate the risk of overfitting or correct optimism in model-performance estimates.

### 2.7 Statistical analysis

No *a priori* power calculation was performed; the analytical sample was determined by data availability. All analyses were executed using R (version 4.4.0) and the Free Statistics platform (version 2.4, Beijing, China; http://www.clinicalscientists.cn/freestatistics). Two-sided tests were applied throughout, with P < 0.05 considered statistically significant.

Continuous variables are reported as mean ± standard deviation (for normally distributed data) or median with interquartile range (for skewed distributions), while categorical variables are expressed as frequencies and percentages. Group comparisons employed one-way analysis of variance, Kruskal–Wallis test, or chi-square test, as dictated by data type and distribution.

Multivariable logistic regression models were adjusted for covariates selected via LASSO regression, including: Model 1 (unadjusted); Model 2 (age, preBMI, Nulliparity); Model 3 (Model 2 + ALT, GGT); Model 4 (Model 3 + HOMA-IR); and Model 5 (Model 4 + TC, LDL, NAFLD—the fully adjusted model).

Restricted cubic spline (RCS) regression with four knots was fitted to explore dose–response relationships and potential nonlinear trends between each exposure and GDM risk, with adjustment identical to Model 5. Sensitivity analyses using alternative knot placement (three knots) were performed to assess the robustness of the dose–response pattern.

In sensitivity analyses, we repeated the primary regression models after excluding participants with NAFLD (n remaining = 471), using the same covariate structure except omitting NAFLD. Subgroup analyses were performed according to age (<35 vs. ≥35 years), parity, preBMI (<25 vs. ≥25 kg/m²), HOMA-IR (≤2 vs. >2), and NAFLD status, with interaction tested via the likelihood ratio test.

Receiver operating characteristic (ROC) curves were used to quantify discrimination. For each individual index, we calculated the AUC, its 95% CI, and the threshold maximizing the Youden J statistic (sensitivity + specificity − 1), with the corresponding sensitivity and specificity. For combinations of indices (AIP + AIP-BMI, AIP + AIP-WC, AIP-BMI + AIP-WC, and all three), multivariable logistic regression models were fitted and their predicted probabilities were used as composite scores; the reported thresholds for these models are therefore probability cutoffs rather than directly interpretable sums of index values. Correlated AUCs were compared using DeLong tests. Continuous net reclassification improvement (NRI) and integrated discrimination improvement (IDI) were used to compare combined panels. All discrimination estimates were obtained from the same data used to fit the models and are therefore apparent, in-sample estimates; no bootstrap optimism correction, calibration assessment, or external validation was performed.

## 3 Results

### 3.1 Baseline characteristics of the study population

The final sample comprised 581 women (**Table 1**), with a mean age of 32.1 ± 3.8 years and mean preBMI of 22.0 ± 3.5 kg/m²; 36 participants (6.2%) developed GDM. Compared with the non-GDM group, the GDM group had higher formula-derived BMI (26.5 vs 22.2 kg/m²), formula-derived WC (92.7 vs 83.2 cm), preBMI (25.8 vs 21.8 kg/m²), ALT (15.0 vs 11.0 IU/L), GGT (17.7 vs 13.7 IU/L), TG (176.1 vs 115.2 mg/dL), HOMA-IR (2.9 vs 1.5), and NAFLD prevalence (55.6% vs 16.5%), and lower HDL-C (58.9 vs 65.3 mg/dL) (all P ≤ 0.006). AIP, AIP-BMI, and AIP-WC were also higher in the GDM group (all P < 0.001).

**Table 1:** Baseline characteristics of participants.

Variables	Total (n = 581)	Non-GDM (n = 545)	GDM (n = 36)	P-value
BMI (kg/m²)	22.4 ± 3.5	22.2 ± 3.2	26.5 ± 5.0	< 0.001
WC (cm)	83.8 ± 9.6	83.2 ± 9.2	92.7 ± 11.1	< 0.001
Age (years)	32.1 ± 3.8	32.0 ± 3.8	32.6 ± 3.6	0.415
preBMI (kg/m²)	22.0 ± 3.5	21.8 ± 3.2	25.8 ± 5.2	< 0.001
Nulliparity				0.964
Yes	304 (52.3)	285 (52.3)	19 (52.8)	
No	277 (47.7)	260 (47.7)	17 (47.2)	
ALT (IU/L)	11.0 (8.0, 15.0)	11.0 (8.0, 14.0)	15.0 (10.0, 25.5)	0.003
GGT (IU/L)	14.0 ± 8.4	13.7 ± 8.3	17.7 ± 8.9	0.005
TC (mg/dl)	172.8 ± 27.1	172.4 ± 26.9	180.2 ± 29.7	0.092
TG (mg/dl)	119.0 ± 47.6	115.2 ± 41.7	176.1 ± 83.1	< 0.001
HDL (mg/dl)	64.9 ± 13.6	65.3 ± 13.3	58.9 ± 16.5	0.006
LDL (mg/dl)	84.0 ± 21.6	84.0 ± 21.1	84.0 ± 28.7	0.996
NAFLD				< 0.001
No	470 (81.0)	454 (83.5)	16 (44.4)	
Yes	110 (19.0)	90 (16.5)	20 (55.6)	
HOMA_IR	1.5 (1.0, 2.3)	1.5 (0.9, 2.2)	2.9 (1.7, 4.7)	< 0.001
AIP	0.2 (0.1, 0.4)	0.2 (0.1, 0.4)	0.4 (0.3, 0.6)	< 0.001
AIP tertile				< 0.001
Q1 (<0.16)	203 (35.0)	200 (36.8)	3 (8.3)	
Q2 (0.16-0.32)	180 (31.0)	173 (31.8)	7 (19.4)	
Q3 (≥0.32)	197 (34.0)	171 (31.4)	26 (72.2)	
AIP_BMI	4.9 (2.3, 8.5)	4.6 (2.3, 7.8)	11.5 (9.0, 14.7)	< 0.001
AIP_WC	18.6 (8.8, 31.0)	17.7 (8.2, 29.3)	40.8 (33.3, 50.1)	< 0.001

Values are expressed as mean ± standard deviation, median (interquartile range), or n (%). AIP, atherogenic index of plasma; BMI, body mass index; preBMI, pre-pregnancy body mass index; WC, waist circumference; ALT, alanine aminotransferase; GGT, gamma-glutamyl transferase; TC, total cholesterol; TG, triglyceride; HDL-C, high-density lipoprotein cholesterol; LDL-C, low-density lipoprotein cholesterol; NAFLD, nonalcoholic fatty liver disease; HOMA-IR, homeostasis model assessment of insulin resistance.

In the analytic sample, formula-derived BMI had a mean of 22.43 ± 3.53 kg/m², a median of 21.83 kg/m², and a range of 15.43–40.90 kg/m²; formula-derived WC had a mean of 83.79 ± 9.63 cm, a median of 83.00 cm, and a range of 54.50–125.00 cm. The mean within-participant difference between formula-derived first-trimester BMI and preBMI was 0.41 ± 1.04 kg/m² (median 0.37 kg/m²). Apart from the single non-positive WC value excluded before analysis, no additional non-positive formula-derived values were observed.

### 3.2 Univariate analysis

In univariate logistic regression (**Table 2**), AIP (per 0.1-unit increase: OR 1.77, 95% CI 1.47–2.12), AIP-BMI (OR 1.30, 95% CI 1.20–1.40), and AIP-WC (OR 1.07, 95% CI 1.05–1.09) were positively associated with GDM (all P < 0.001). Significant crude associations were also observed for preBMI, WC, ALT, GGT, TG, HOMA-IR, and NAFLD.

**Table 2:** Results of univariate analysis.

Variable	OR (95% CI)	P-value
BMI (kg/m²)	1.3 (1.2-1.42)	<0.001
WC (cm)	1.09 (1.05-1.12)	<0.001
Age (years)	1.04 (0.95-1.14)	0.414
preBMI (kg/m²)	1.27 (1.17-1.38)	<0.001
Nulliparity	0.98 (0.5-1.93)	0.964
ALT (IU/L)	1.04 (1.01-1.06)	0.002
GGT (IU/L)	1.03 (1.01-1.06)	0.011
TC (mg/dl)	1.01 (1-1.02)	0.093
TG (mg/dl)	1.02 (1.01-1.02)	<0.001
HDL (mg/dl)	0.96 (0.94-0.99)	0.006
LDL (mg/dl)	1 (0.98-1.02)	0.996
NAFLD	6.31 (3.15-12.64)	<0.001
HOMA_IR	1.49 (1.23-1.8)	<0.001
AIP*10	1.77 (1.47-2.12)	<0.001
AIP_BMI	1.30 (1.20–1.40)	<0.001
AIP_WC	1.07 (1.05–1.09)	<0.001

*AIP is scaled per 0.1-unit.

### 3.3 Multivariable associations of AIP, AIP-BMI, and AIP-WC with GDM

AIP, AIP-BMI, and AIP-WC remained positively associated with GDM across the sequential models (**Table 3**). In Model 5, each 0.1-unit increase in AIP was associated with an OR of 1.66 (95% CI 1.36–2.03; P < 0.001), and Q3 versus Q1 was associated with an OR of 7.86 (95% CI 1.93–32.08; P for trend = 0.001). The corresponding per-unit ORs were 1.20 (95% CI 1.11–1.31; P < 0.001) for AIP-BMI and 1.05 (95% CI 1.03–1.08; P < 0.001) for AIP-WC.

**Table 3:** Associations of AIP and its modified indices with GDM in sequential logistic regression models.

Variable	AIP*10		AIP tertile				AIP-BMI		AIP-WC	
	OR (95% CI)	P-value	Q1 (OR, 95% CI)	Q2 (OR, 95% CI)	Q3 (OR, 95% CI)	P-trend	OR (95% CI)	P-value	OR (95% CI)	P-value
Model 1	1.77 (1.47-2.12)	<0.001	1(Ref)	2.7 (0.69-10.59)	10.14 (3.02-34.06)	<0.001	1.30 (1.20–1.40)	<0.001	1.07 (1.05–1.09)	<0.001
Model 2	1.76 (1.47-2.12)	<0.001	1(Ref)	2.67 (0.68-10.51)	10.06 (2.99-33.9)	<0.001	1.23 (1.14–1.33)	<0.001	1.06 (1.04–1.08)	<0.001
Model 3	1.67 (1.37-2.03)	<0.001	1(Ref)	1.83 (0.44-7.52)	6.72 (1.91-23.68)	<0.001	1.23 (1.13–1.33)	<0.001	1.06 (1.03–1.08)	<0.001
Model 4	1.66 (1.36-2.03)	<0.001	1(Ref)	2.29 (0.5-10.49)	7.71 (1.96-30.35)	<0.001	1.22 (1.13–1.33)	<0.001	1.06 (1.03–1.08)	<0.001
Model 5	1.66 (1.36-2.03)	<0.001	1(Ref)	2.84 (0.6-13.47)	7.86 (1.93-32.08)	0.001	1.20 (1.11–1.31)	<0.001	1.05 (1.03–1.08)	<0.001

Model 1: Unadjusted.

Model 2: Adjusted for age, preBMI, and nulliparity.

Model 3: Model 2 + ALT and GGT.

Model 4: Model 3 + HOMA-IR.

Model 5: Model 4 + TC, LDL-C, and NAFLD.

AIP was scaled per 0.1-unit; AIP-BMI and AIP-WC were modeled per 1-unit increase.

**Figure 2:**
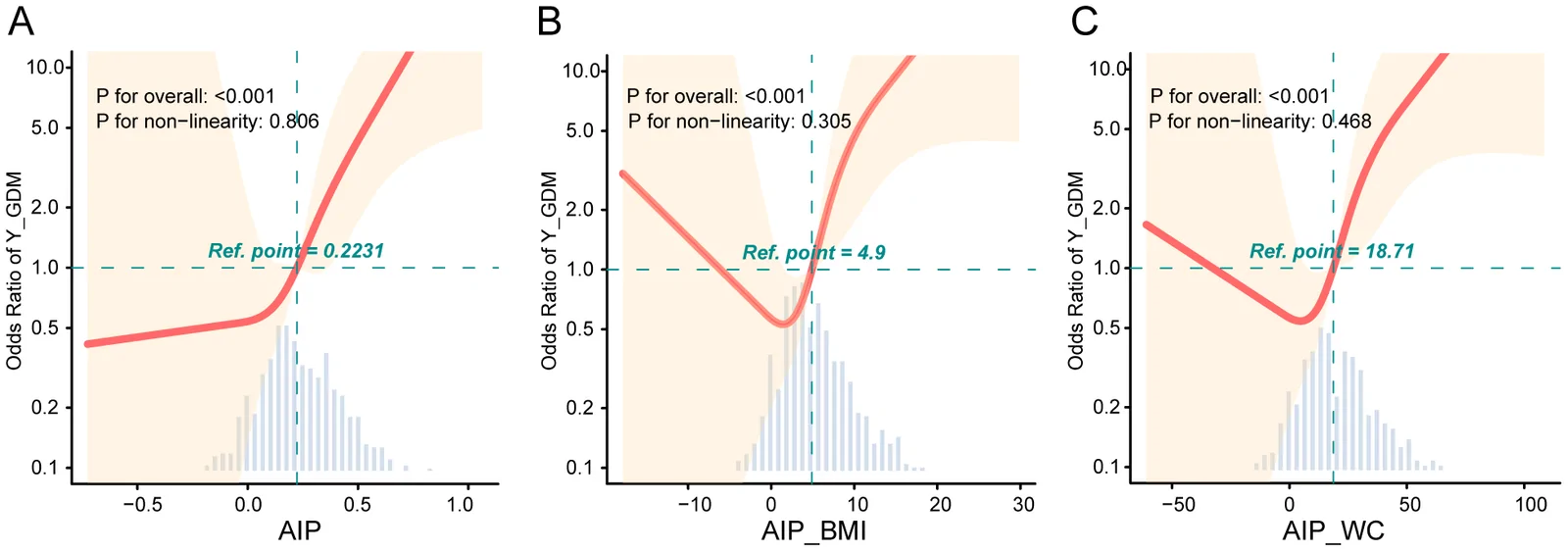
Restricted cubic spline associations of **(A)** AIP, **(B)** AIP-BMI, and **(C)** AIP-WC with GDM. Models were adjusted for age, preBMI, nulliparity, ALT, GGT, HOMA-IR, TC, LDL-C, and NAFLD. Histograms show exposure distributions; dashed lines indicate the reference values and OR = 1.

Restricted cubic spline analyses with four knots showed significant overall associations for AIP, AIP-BMI, and AIP-WC (all P for overall <0.001), but no statistical evidence of nonlinearity (P = 0.806, 0.305, and 0.468, respectively; **Figure 2**). The AIP-BMI and AIP-WC curves displayed an apparent low-end dip before increasing, but the confidence bands were wide at the distributional extremes. Sensitivity analyses using three knots gave similar results (P for nonlinearity = 0.791, 0.893, and 0.993, respectively; **Supplementary Figure 1**). Accordingly, the low-end shape should be regarded as an unstable descriptive feature rather than evidence that very low AIP confers excess risk.

### 3.4 Sensitivity analysis

After exclusion of participants with NAFLD (n = 471), all three indices remained associated with GDM (**Table 4**). In the fully adjusted sensitivity model, the ORs were 1.79 (95% CI 1.34–2.38) per 0.1-unit increase in AIP, 6.12 (95% CI 1.31–28.71) for Q3 versus Q1, 1.28 (95% CI 1.13–1.46) per unit of AIP-BMI, and 1.07 (95% CI 1.04–1.11) per unit of AIP-WC (all P < 0.001 except P for trend = 0.004).

**Table 4:** Sensitivity analysis after excluding participants with NAFLD.

Variable	AIP*10		AIP tertile				AIP-BMI		AIP-WC	
	OR (95% CI)	P-value	Q1 (OR, 95% CI)	Q2 (OR, 95% CI)	Q3 (OR, 95% CI)	P-trend	OR (95% CI)	P-value	OR (95% CI)	P-value
Model 1	1.78 (1.36-2.33)	<0.001	1(Ref)	0.5 (0.04-5.57)	7 (1.55-31.54)	0.003	1.29 (1.15–1.45)	<0.001	1.07 (1.04–1.11)	<0.001
Model 2	1.77 (1.35-2.32)	<0.001	1(Ref)	0.47 (0.04-5.3)	6.7 (1.48-30.32)	0.003	1.29 (1.14–1.46)	<0.001	1.07 (1.04–1.11)	<0.001
Model 3	1.82 (1.37-2.42)	<0.001	1(Ref)	0.46 (0.04-5.11)	6.42 (1.41–29.3)	0.003	1.29 (1.14–1.47)	<0.001	1.07 (1.04–1.11)	<0.001
Model 4	1.81 (1.36-2.41)	<0.001	1(Ref)	0.39 (0.03-4.85)	5.87 (1.27–27.06)	0.004	1.29 (1.14–1.46)	<0.001	1.07 (1.04–1.11)	<0.001
Model 5	1.79 (1.34-2.38)	<0.001	1(Ref)	0.41 (0.03-5.07)	6.12 (1.31–28.71)	0.004	1.28 (1.13–1.46)	<0.001	1.07 (1.04–1.11)	<0.001

The sensitivity analysis included 471 participants without NAFLD and used the same model sequence as **Table 3**, except that NAFLD was omitted from Model 5.

Model 5 adjusted for age, preBMI, nulliparity, ALT, GGT, HOMA-IR, TC, and LDL-C.

### 3.5 Subgroup analysis

**Figure 3:**
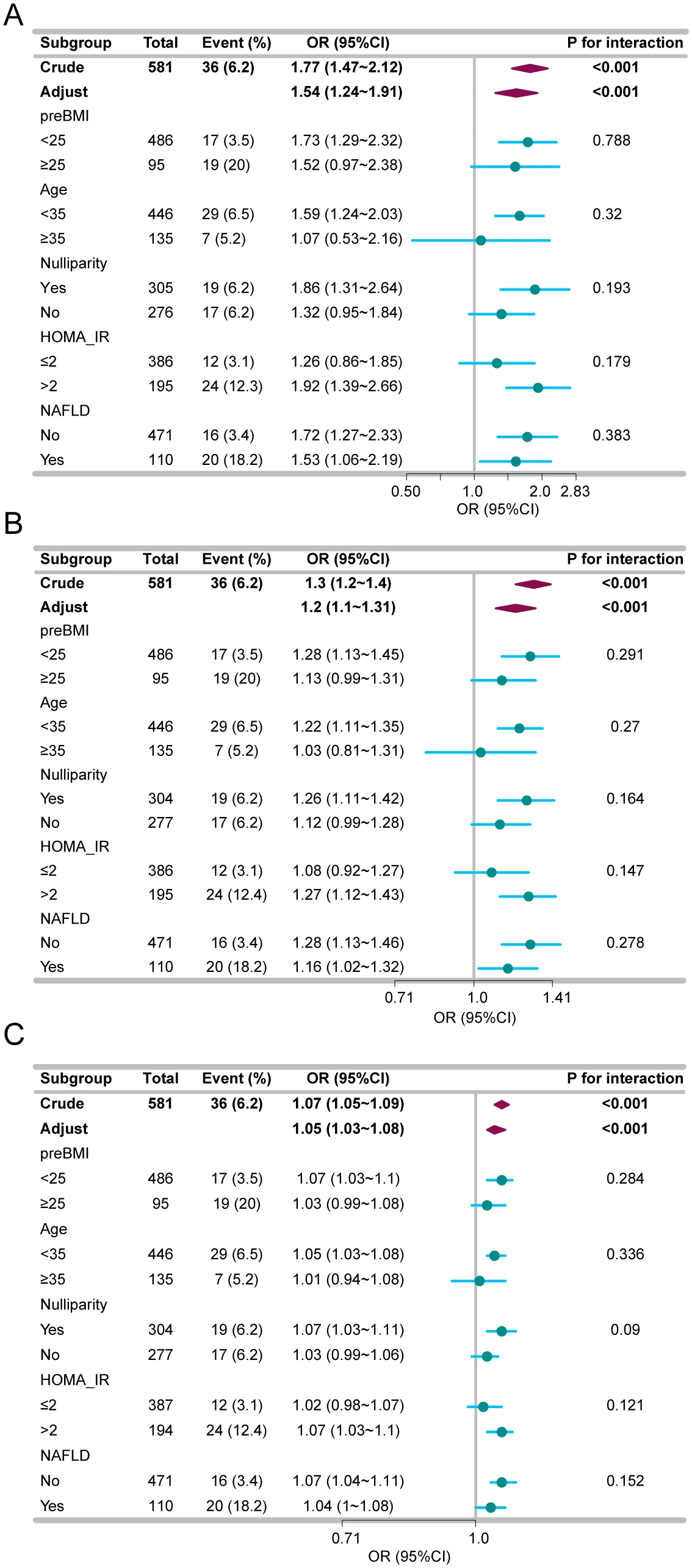
Forest plots for **(A)** AIP per 0.1-unit, **(B)** AIP-BMI per 1-unit, and **(C)** AIP-WC per 1-unit in prespecified and exploratory subgroups. Each stratified model was adjusted for all Model 5 covariates except the stratification factor. P values in subgroup rows are interaction tests.

No interaction was statistically detected across strata defined by preBMI, age, nulliparity, HOMA-IR, or NAFLD status (all P for interaction >0.05; **Figure 3**). Because several strata contained few GDM events, these null interaction tests should not be interpreted as evidence of homogeneous effects.

### 3.6 Predictive performance of AIP and its modified indices

**Figure 4:**
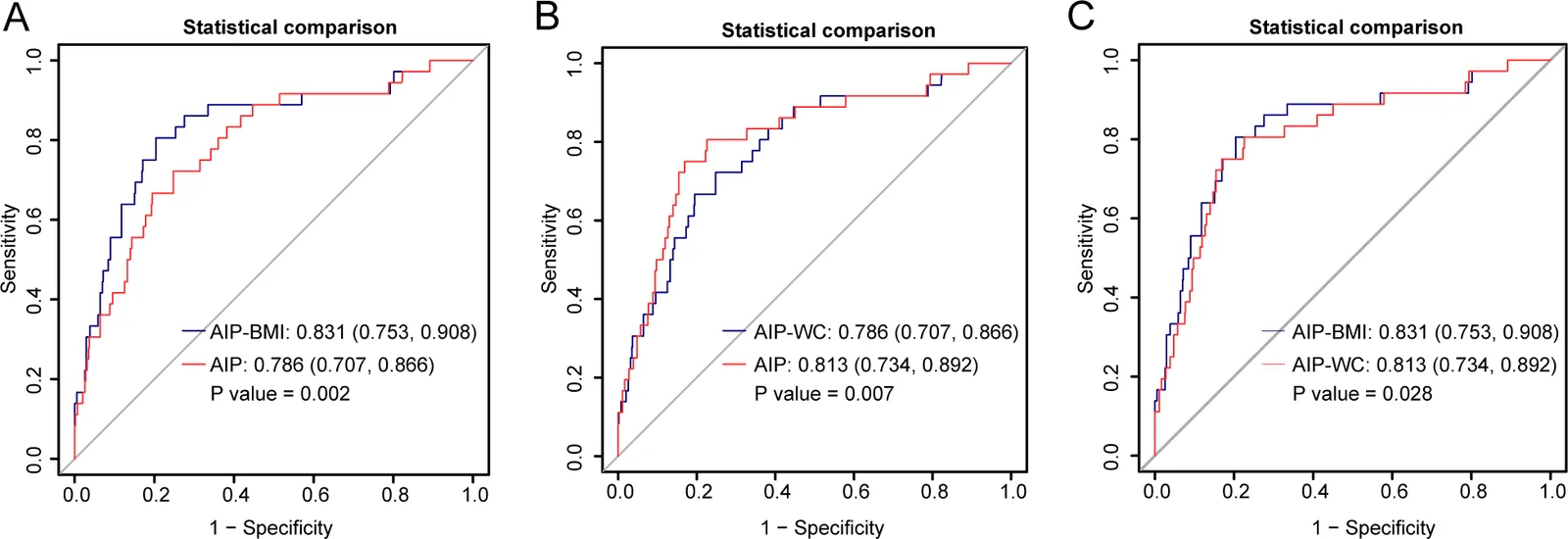
Pairwise DeLong comparisons of ROC curves for **(A)** AIP-BMI versus AIP, **(B)** AIP-WC versus AIP, and **(C)** AIP-BMI versus AIP-WC.

The apparent discriminatory performance of the individual indices and their combinations is summarized in **Table 5** and **Figure 4** and **Supplementary Figure 2**. AIP-BMI had an AUC of 83.05% (95% CI 75.30%–90.81%), sensitivity of 81%, and specificity of 80%; AIP-WC had an AUC of 81.32% (95% CI 73.45%–89.19%); and AIP had an AUC of 78.66% (95% CI 70.71%–86.61%). DeLong comparisons showed higher AUCs for AIP-BMI versus AIP (P = 0.002), AIP-WC versus AIP (P = 0.007), and AIP-BMI versus AIP-WC (P = 0.028) (**Figure 4**). The AIP + AIP-BMI model had the largest apparent AUC among evaluated models (84.19%, 95% CI 76.36%–92.02%; sensitivity 81%, specificity 81%). Compared with AIP + AIP-WC, AIP + AIP-BMI yielded an NRI of 0.1889 (95% CI 0.0376–0.3403; P = 0.014) and an IDI of 0.0415 (95% CI 0.0053–0.0777; P = 0.025) (**Table 6**). Comparisons with AIP-BMI + AIP-WC were not significant (NRI P = 0.067; IDI P = 0.357), and adding AIP-WC to AIP + AIP-BMI did not improve reclassification (NRI P = 0.157; IDI P = 0.562). These are exploratory, in-sample estimates and were not optimism-corrected or externally validated.

**Table 5:** Apparent discrimination of individual indices and combined models for GDM.

Variable	AUC (95% CI)	Best threshold	Specificity	Sensitivity	Youden index
AIP	78.66% (70.71%–86.61%)	0.3557	0.75	0.72	0.47
AIP-BMI	83.05% (75.30%–90.81%)	8.6372	0.8	0.81	0.61
AIP-WC	81.32% (73.45%–89.19%)	34.0492	0.83	0.75	0.58
AIP+AIP-BMI	84.19% (76.36%–92.02%)	0.0567	0.81	0.81	0.62
AIP+AIP-WC	82.84% (75.02%–90.66%)	0.0681	0.82	0.78	0.60
AIP-BMI+AIP-WC	83.58% (75.82%–91.33%)	0.0456	0.73	0.89	0.62
AIP+AIP-BMI+AIP-WC	84.16% (76.34%–91.98%)	0.0551	0.8	0.81	0.61

AUCs and optimal thresholds are apparent estimates from the model-development sample. The Youden J statistic is sensitivity + specificity − 1. Thresholds for combined models are predicted-probability cutoffs and require external validation.

**Table 6:** Continuous NRI and IDI comparisons for combined models.

Model comparison	NRI (95% CI)	P value	IDI (95% CI)	P value
AIP + AIP-BMI vs AIP + AIP-WC	0.1889 (0.0376–0.3403)	0.014	0.0415 (0.0053–0.0777)	0.025
AIP + AIP-BMI vs AIP-BMI + AIP-WC	0.0852 (−0.0059–0.1762)	0.067	0.0081 (−0.0091–0.0253)	0.357
AIP + AIP-BMI vs AIP + AIP-BMI + AIP-WC	0.0037 (−0.0014–0.0088)	0.157	−0.0009 (−0.0039–0.0021)	0.562

## 4 Discussion

In this secondary analysis of 581 Korean women with singleton pregnancies, we examined the associations of first-trimester AIP, AIP-BMI, and AIP-WC with subsequent GDM and compared their apparent discriminatory performance. Three principal findings emerged. First, all three indices were associated with GDM after multivariable adjustment. Second, AIP-BMI had the largest apparent AUC among the individual indices. Third, the AIP + AIP-BMI model had the largest apparent AUC among the evaluated combined models. These findings support further evaluation of obesity-integrated AIP indices but do not establish a validated clinical prediction tool.

The association between elevated AIP and GDM remained after adjustment for age, preBMI, nulliparity, ALT, GGT, HOMA-IR, TC, LDL-C, and NAFLD, consistent with the earlier analysis of the same cohort by Zhang et al. (14). Elevated AIP reflects an atherogenic lipid pattern linked to insulin resistance (11, 20). Remnant cholesterol may also contribute to GDM through oxidative stress and insulin resistance pathways (21). Pregnancy-related insulin resistance may amplify the metabolic consequences of dyslipidemia (22, 23). Because this study is observational, these mechanisms should be regarded as interpretive rather than causal.

Both modified indices showed higher apparent AUCs than AIP alone. AIP-BMI had an apparent AUC of 83.05%, compared with 81.32% for AIP-WC and 78.66% for AIP; pairwise DeLong P values were 0.002, 0.007, and 0.028, respectively. In fully adjusted models, the corresponding per-unit ORs were 1.20 (95% CI 1.11–1.31) for AIP-BMI and 1.05 (95% CI 1.03–1.08) for AIP-WC. These findings suggest that combining AIP with measures of adiposity may improve discrimination in this dataset. A maternal lipid profile characterized by elevated triglycerides and reduced HDL-C has been consistently associated with GDM in large meta-analyses (24), and early-pregnancy metabolic biomarkers may improve early detection (25). However, BMI and WC were formula-derived rather than directly measured, so the relative performance of AIP-BMI and AIP-WC requires confirmation using contemporaneous anthropometric measurements.

When indices were evaluated in combination, the AIP + AIP-BMI model had the largest apparent AUC (84.19%; sensitivity 81%, specificity 81%), approximately 5.5 percentage points above AIP alone. It showed significant NRI and IDI relative to AIP + AIP-WC, but not relative to AIP-BMI + AIP-WC, and adding all three indices did not improve reclassification. Thus, AIP + AIP-BMI was the most parsimonious high-performing model in this dataset; however, all estimates were obtained in the model-development sample without optimism correction or external validation and should not be interpreted as confirmed clinical superiority.

The NAFLD-excluded sensitivity analysis yielded associations in the same direction. No subgroup interaction was statistically detected across age, nulliparity, preBMI, HOMA-IR, or NAFLD strata, but several strata contained few GDM events. These analyses therefore support the consistency of the overall direction but do not establish homogeneous effects across subgroups.

The findings have potential research implications for early GDM risk assessment because AIP and BMI can be calculated from commonly available fasting lipid and anthropometric measurements. In this dataset, however, BMI and WC were formula-derived and the models were developed and evaluated in the same sample. Accordingly, these indices should be regarded as candidates for further validation rather than as stand-alone screening thresholds or replacements for guideline-recommended GDM testing.

Several methodological considerations should be acknowledged. As a secondary analysis, information on diet, physical activity, and family history of diabetes was unavailable, and residual confounding cannot be excluded. BMI and WC were reconstructed algebraically from full-precision HSI and FLI components rather than measured directly. Formula-derived BMI was close to preBMI at the group and within-participant levels, but the FLI inversion becomes increasingly sensitive to FLI error near zero; one participant with a formula-derived WC of 0 cm was excluded, and no direct WC measurement was available for validation. Only 36 GDM events were available for a fully adjusted model containing the exposure and nine covariates, yielding an events-per-parameter ratio of approximately 3.6. LASSO variable selection does not remove risks of coefficient instability and overfitting; the three events in AIP Q1 and the wide Q3 confidence interval (1.93–32.08) illustrate this imprecision. AUCs, thresholds, NRI, and IDI were estimated in the model-development sample without bootstrap optimism correction, calibration assessment, or external validation and must therefore be regarded as apparent estimates. Subgroup analyses were underpowered, and nonsignificant interaction tests do not confirm homogeneous effects. Finally, measurements were obtained at one early-pregnancy time point in Korean women, and use of the two-step ACOG approach limits generalizability to populations diagnosed with different criteria.

## 5 Conclusion

In summary, first-trimester AIP, AIP-BMI, and AIP-WC were associated with subsequent GDM. AIP-BMI had the largest apparent AUC among the individual indices, and AIP + AIP-BMI was the most parsimonious high-performing combined model in this dataset. These findings are hypothesis-generating and require confirmation in larger cohorts with directly measured BMI and WC and appropriate validation.

## Data Availability

The original contributions presented in the study are included in the article/**Supplementary Material**. Further inquiries can be directed to the corresponding author.

## Supplementary material

Supplementary Figure 1 Sensitivity analysis of restricted cubic spline associations for **(A)** AIP, **(B)** AIP-BMI, and **(C)** AIP-WC using three knots and Model 5 covariate adjustment.

Supplementary Figure 2 ROC curves for BMI, WC, AIP, AIP-BMI, AIP-WC, and the evaluated combined models. Curves and AUCs are apparent estimates from the model-development sample.
